# Role of IL‐18 in atopic asthma is determined by balance of IL‐18/IL‐18BP/IL‐18R

**DOI:** 10.1111/jcmm.13323

**Published:** 2017-09-18

**Authors:** Huiyun Zhang, Junling Wang, Ling Wang, Hua Xie, Liping Chen, Shaoheng He

**Affiliations:** ^1^ Translational Medicine Institute Shenyang Medical College Shenyang Liaoning China; ^2^ Allergy and Clinical Immunology Research Centre the First Affiliated Hospital of Jinzhou Medical University Jinzhou Liaoning China; ^3^ The PLA Center of Respiratory and Allergic Disease Diagnosing Management General Hospital of Shenyang Military Area Command Shenyang China

**Keywords:** IL‐18, IL‐18BP, IL‐18R, monocyte, B cell, asthma

## Abstract

It is recognized that IL‐18 is related to development of asthma, but role of IL‐18 in asthma remains controversial and confusing. This is largely due to lack of information on expression of IL‐18 binding protein (BP) and IL‐18 receptor (R) in asthma. In this study, we found that plasma levels of IL‐18 and IL‐18BP were elevated in asthma. The ratio between plasma concentrations of IL‐18 and IL‐18BP was 1:12.8 in asthma patients. We demonstrated that 13‐fold more monocytes, 17.5‐fold more neutrophils and 4.1‐fold more B cells express IL‐18BP than IL‐18 in asthmatic blood, suggesting that there is excessive amount of IL‐18BP to abolish actions of IL‐18 in asthma. We also discovered that more IL‐18R+ monocytes, neutrophils and B cells are located in asthmatic blood. Once injected, IL‐18 eliminated IL‐18R+ monocytes in blood, but up‐regulated expression of IL‐18R in lung macrophages of OVA‐sensitized mice. Our data clearly indicate that the role of IL‐18 in asthma is very likely to be determined by balance of IL‐18/IL‐18BP/IL‐18R expression in inflammatory cells. Therefore, IL‐18R blocking or IL‐18BP activity enhancing therapies may be useful for treatment of asthma.

## Introduction

Interleukin (IL)‐18 is a product of the inflammasome, which is involved in host defence against viral and bacterial stimuli by modulating the immune response 1. Recently, however, evidence has accumulated that IL‐18 expression is increased in many presentations of allergic disease 2. In particular, it is required for differentiation of regulatory T cells and protection against allergic asthma 3. It was observed that IL‐18 and IL‐18 receptor (IL‐18R) were strongly expressed in the lungs of fatal asthma 4, serum IL‐18 levels were significantly high in asthmatic children 5 and IL‐18 variants were significantly associated with asthma severity 6, implicating that IL‐18 likely contributes to the development of asthma.

IL‐18R is part of the IL‐1R/TLR superfamily signalling *via* a MyD88‐dependent pathway. A wide range of cells including Th1 cells 7, natural killer cells, natural killer T cells, mast cells and basophils expresses the IL‐18R 8.

IL‐18 (BP) is an endogenous antagonist with high neutralizing capacity that inhibits the action of IL‐18 by preventing interaction with its cell surface receptors 9. At a molar excess of two, IL‐18BP neutralizes IL‐18 to >95% 10. An imbalance between IL‐18 and IL‐18BP expression may account for increased IL‐18 activity in asthma. However, little is known about expression of IL‐18BP and IL‐18R in monocytes, neutrophils and B cells in asthma.

It has been reported that *Alternaria* extract induced rapid release of IL‐18 from cultured normal human bronchial epithelial cells and directly initiated Th2 differentiation 11, and that IL‐18 can induce release of IFN‐γ, IL‐13 and eotaxin in the lungs of ovalbumin‐sensitized and challenged transgenic mice along with an airway hyper‐responsiveness 12, suggesting that allergens may cause allergic airway disorders through IL‐18‐related mechanisms. However, little is known about influence of allergens on expression of IL‐18, IL‐18BP and IL‐18R in asthma.

The findings that increased release of IL‐18 by airway macrophages is associated with both the acute and chronic forms of hypersensitivity pneumonitis 13, that IL‐18 is located in neutrophils 14 and facilitates neutrophil transmigration 15, that IL‐18 down‐regulates B‐cell migration 16, and that virus antigen EBNA2 induces expression of IL‐18R in B cells 17 implicate that macrophage, neutrophils and B cells may participate in the development of airway inflammatory disorders including asthma *via* a IL‐18‐related mechanism. Therefore, the aim of this study is to investigate the expression of IL‐18, IL‐18BP and IL‐18R in inflammatory cells of atopic asthma, and influence of allergens on their expression.

## Materials and methods

### Reagents

The following reagents were purchased from Biolegend (San Diego, CA, USA): red blood cell lysis buffer, PE/Cy7‐conjugated mouse anti‐human CD14 antibody, PerCP‐conjugated mouse anti‐human CD16 antibody, APC/Cy7‐conjugated mouse anti‐human CD19 antibody, APC/Cy7‐conjugated rat antimouse Ly‐6G/Ly‐6C antibody, Brilliant Violet (BV) 421‐conjugated rat antimouse CD11b antibody, APC/Cy7‐conjugated Armenian Hamster antimouse CD11c antibody, PE/Cy7‐conjugated rat antimouse F4/80 antibody, BV510‐conjugated donkey anti‐rabbit IgG polyclonal antibody, Zombie Aqua™ Fixable Viability Kit, Zombie Green™ Fixable Viability Kit, human Fc receptor blocking solution, antimouse CD16/32 antibody and brefeldin A. FITC‐conjugated rabbit antimouse IL‐18BP polyclonal antibody was from Cloud clone (Houston, TX, USA). Recombinant mouse IL‐18 protein, APC‐conjugated mouse anti‐human IL‐18Rα antibody, PE‐conjugated mouse anti‐human IL‐18 antibody, APC‐conjugated rat antimouse IL‐18R antibody and their isotype antibodies: mouse IgG1 APC‐conjugated antibody, mouse IgG1 PE‐conjugated antibody and rat IgG1 APC‐conjugated antibody were supplied by R&D Systems (Minneapolis, MN, USA). Rabbit anti‐human IL‐18BP antibody and its isotype antibody rabbit IgG were obtained from Abcam (Cambridge, UK). Ovalbumin (OVA, Grade V), trypan blue dye, collagenase, hyaluronidase and DNase were purchased from Sigma‐Aldrich (St Louis, MO, USA). Cytofix/Cytoperm™ Fixation/Permeabilization Kit was bought from BD Biosciences Pharmigen (Beldford, MA, USA). Human IL‐18BP and human IL‐18 ELISA kit were bought from ImmunoWay Biotechnology Company (Newark, DE, USA) and ExCell Bio (Shanghai, China), respectively. Foetal bovine serum (FBS, Hyclone) and RPMI 1640 were purchased from Gibco BRL (Grand Island, NY, USA). *Artemisia sieversiana* wild allergen extract (ASWE), *Dermatophagoide* allergen extract (DAE) or *Platanus* pollen allergen extract (PPAE) were offered by Macro Union Pharmaceutical Co. Ltd (Beijing, China). Allergens for skin prick tests were supplied by ALK‐Abelló, Inc. (Denmark). Most of the general‐purpose chemicals such as salts and buffer components were of analytical grade.

### Volunteers and animals

A total of 31 patients with asthma and 14 healthy control (HC) volunteer were recruited in the study. Their general characteristics were summarized in Table 1. The diagnosing criteria of asthma were conformed to the Global Initiative for Asthma 18. All mild asthmatic patients were asked to stop anti‐allergy medication for at least 2 weeks prior to attending the study (those could not stop anti‐allergy drugs were excluded). The recruited patients did not have any airway infection more than one month.

**Table 1:** Characteristics of adult volunteers

Population	Case	Age (year)	Female/male	History (year)	Onset age (year)
HC	14	25 (22–28)	9/5	0	0
Asthma	31	35 (18–65)	20/11	3.5 (0–30)	36 (18–57)
Cockroach (+)	2	24 (20–28)	1/1	2 (1–3)	22 (19–25)
Mite (+)	16	40 (18–65)	10/6	10 (0.5–30)	33 (18–35)
Grass pollen (+)	7	32 (18–52)	5/2	3.5 (1–6)	26 (16–48)
Tree pollen (+)	6	28 (24–60)	4/2	2.5 (0–4.5)	39 (23–57)

Median values (range) are shown. Specific allergens were examined by skin prick test. HC = healthy control.

The informed consent from each volunteer according to the declaration of Helsinki and agreement with the Ethical Committee of the Second Affiliated Hospital of Shenyang Medical College, or with the General Hospital of Shenyang Military Region of PLA, or with the First Affiliated Hospital of Jinzhou Medical University was obtained (Trial registry: Chinese clinical trial; registration number: ChiCTR‐BOC‐16010279).

Immediately after admission (acute exacerbation stage), the blood from each patient with asthma was collected. Blood from HCs was collected in the outpatient clinic. From each individual, 10 ml of peripheral blood was taken into an EDTA containing tube before centrifugation at 450 × *g* for 10 min. The cells were used for flow cytometric analysis, and plasma was collected and frozen at −80°C until use.

Female BALB/c mice (4–6 w, 18–22 g) were obtained from Vital River Laboratory Animal Technology Co. Ltd (Beijing, China), Certificate No 11400700118760. The animals were bred and reared under strict ethical conditions according to international recommendations. They were housed in the Animal Experimental Center of the First Affiliated Hospital of Jinzhou Medical University in a specific pathogen‐free environment with free access to standard rodent chow and water, at a constant temperature 23–28°C and relative humidity of 60–75%. The animal experiment procedures were approved by the Animal Care Committee at Jinzhou Medical University.

### Flow cytometry analysis of IL‐18, IL‐18BP and IL‐18R in human peripheral blood monocytes, neutrophils and B cells

To detect expression of IL‐18, IL‐18BP and IL‐18R in human blood monocytes, neutrophils and B cells, blood cells were stimulated with or without ASWE, DAE or PPAE (all at a concentration of 1.0 μg/ml) for 1 hr at 37°C, respectively, and 2 μg/ml brefeldin A was also added into the tube. Cells were then incubated with human Fc receptor blocking solution and a live/dead cell dye (Zombie Green™ Fixable Viability Kit 19 for 15 min., and each labelled monoclonal antibody including PE/Cy7‐conjugated anti‐human CD14, PerCP‐conjugated anti‐human CD16, APC/Cy7‐conjugated anti‐human CD19 and APC‐conjugated anti‐human IL‐18Rα was added into the tube. After red blood cells being lysed, resuspended leucocytes were fixed and permeabilized using Cytofix/Cytoperm™ Fixation/Permeabilization Kit according to the manufacturer's instructions. This was followed by adding PE‐conjugated anti‐human IL‐18 and anti‐human IL‐18BP primary antibodies into the tube and incubated at 4°C for 30 min. BV510‐conjugated donkey anti‐rabbit polyclonal antibody was added into each tube for 30 min. at room temperature. Finally, cells were resuspended in fluorescence‐activated cell sorting (FACS)‐Flow solution and analysed with FACS Verse flow cytometer (BD Biosciences, San Jose, CA). A total of 10,000 events in live cell gate were analysed for each sample. Data were analysed with FlowJo software version 7.0 (Treestar, Ashland, OR, USA). Dead cells and doublets were excluded from analysis by live/dead cell dyes.

### Mouse sensitization and challenge

Mice were sensitized on days 0, 7, 14 and 21 with an subcutaneous multi‐point injection of 50 μg OVA and 1.5 mg of Al (OH)_3_ suspended in NS to a total volume of 0.5 ml. Non‐sensitized control animals received only the equal volume (0.5 ml) of NS on the same days. On day 25, sensitized mice were challenged with intratracheal injection of 0.1 ml of 5% OVA solution with or without 10 ng/ml of IL‐18 for 3 hrs. After being killed, blood from mice was collected. The cells were used for flow cytometric analysis, and plasma was collected and frozen at −80°C until use. Lung tissues were excised and digested into dispersed cells as described previously 20.

### Flow cytometry analysis of IL‐18BP and IL‐18R in mouse blood monocytes and neutrophils, and lung macrophages

To detect IL‐18BP and IL‐18R expression on monocytes and neutrophils, cells were incubated with antimouse CD16/32, and a live/dead cell dye (Zombie Aqua™ Fixable Viability Kit) 21 for 15 min. Each labelled monoclonal antibody including APC/Cy7‐conjugated antimouse Ly‐6G/Ly‐6C 22, BV421‐conjugated antimouse CD11b and APC‐conjugated antimouse IL‐18R was added into tubes for 15 min. Red blood cells were lysed, and leucocytes were fixed and permeabilized using Cytofix/Cytoperm™ Fixation/Permeabilization Kit according to the manufacturer's instructions. FITC‐conjugated antimouse IL‐18BP was then added into each tube. Finally, cells were processed as for human blood samples and analysed by flow cytometer.

To detect IL‐18BP and IL‐18R expression in macrophages, dispersed cells were incubated with antimouse CD16/32 and a live/dead cell dye (Zombie Aqua™ Fixable Viability Kit) 21 for 15 min. APC/Cy7‐conjugated antimouse CD11c, PE/Cy7‐conjugated antimouse F4/80 and APC‐conjugated antimouse IL‐18R antibodies were then added into the tube for 15 min. Cells were finally processed and analysed as above.

### Determination of levels of IL‐18 and IL‐18BP

Levels of IL‐18 and IL‐18BP in human plasma were measured using ELISA kits according to the manufacturer's instruction.

### Statistics

Statistical analyses were performed using SPSS software (Version 17.0; IBM Corporation, Armonk, NY, USA). Data for expression of IL‐18, IL‐18BP and IL‐18R in leucocytes are displayed as a boxplot, which indicates the median, interquartile range, the largest and smallest values for the number of experiments indicated. Plasma levels of IL‐18 and IL‐18BP are presented as scatter plot. Kruskal–Wallis analysis indicated significant differences between groups, and for the pre‐planned comparisons of interest, the paired Mann–Whitney *U*‐test was employed. For all analyses, *P* < 0.05 was taken as significant.

## Results

### Elevated levels of IL‐18 and IL‐18BP in asthmatic plasma

**Figure 1:**
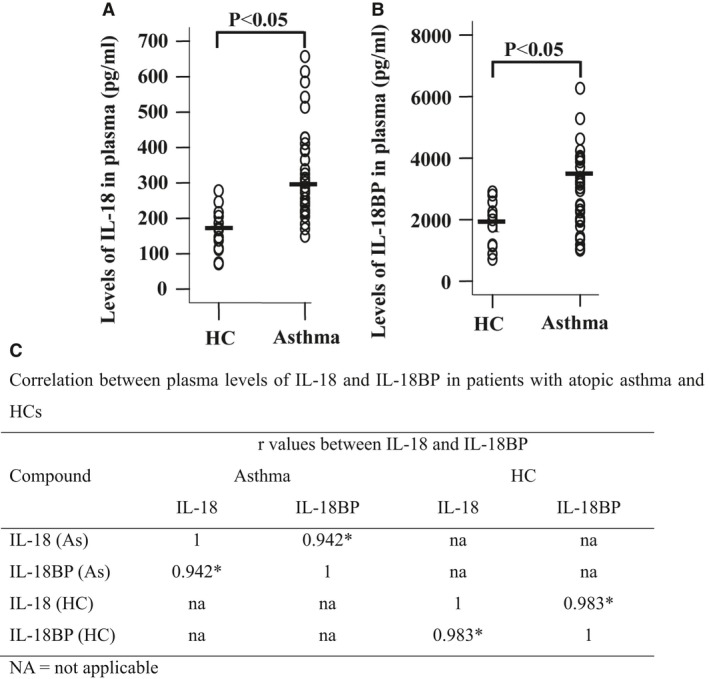
Scatter plots of levels of IL‐18 (**A**) and IL‐18BP (**B**) in plasma of asthmatic and healthy control (HC) volunteers. Each symbol represents the value from 1 volunteer. The median value is indicated with a horizontal line. *P* < 0.05 was taken as statistically significant. (**C**) The spearman's rho/correlation coefficient between the plasma level of IL‐18 and IL‐18BP. * *P* < 0.05.

Serum IL‐18 level was found significantly higher in children who had asthma 5, but the plasma/serum level of IL‐18BP in asthma, and correlation between IL‐18 and IL‐18BP has not been investigated. Using ELISA kits, we observed that levels of IL‐18 were 270.7 and 169.0 pg/ml (Fig. 1A), and levels of IL‐18BP were 3460 and 1910 pg/ml (Fig. 1B) in the plasma of patients with asthma and HC volunteers, respectively. The ratio between plasma concentrations of IL‐18 and IL‐18BP was 1:12.8 in asthmatic patients. It was observed that IL‐18 and IL‐18BP were correlated well with each other in the plasma of patients with asthma and HC volunteers (Fig. 1C).

### Enhanced CD14^+^ monocytes and IL‐18R^+^ monocytes in peripheral blood of patients with atopic asthma

**Figure 2:**
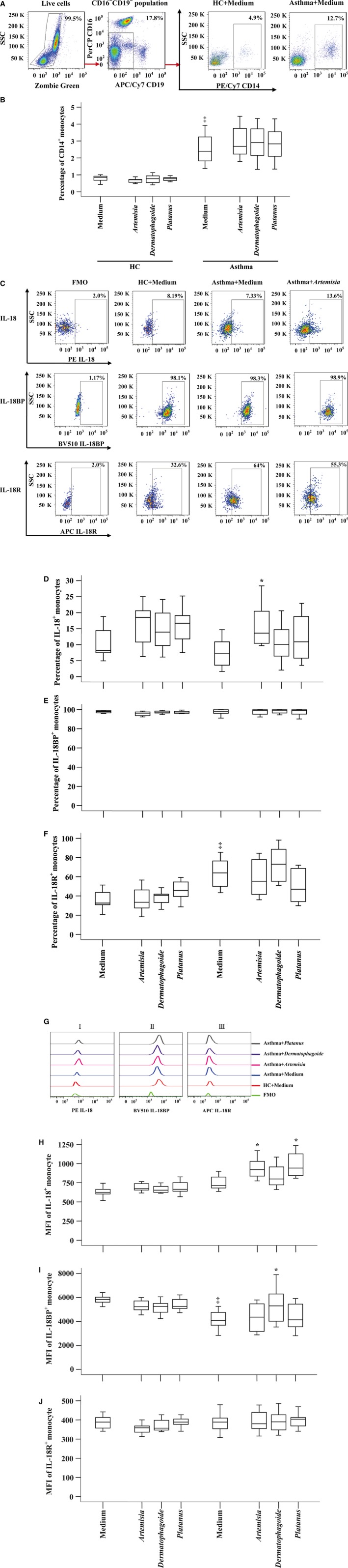
Expression of IL‐18, IL‐18BP and IL‐18 receptor (R) in peripheral blood CD14^+^ monocytes of asthma patients and healthy control (HC) volunteers in the presence or absence of *Artemisia*,* Dermatophagoide* and *Platanus* allergens. (**A**) represents a gating strategy of CD14^+^ monocytes in leucocytes. (**B**) shows proportion of CD14^+^ monocytes in human peripheral blood leucocytes. (**C**) is a gating strategy of expression of IL‐18, IL‐18BP and IL‐18R in CD14^+^ monocytes. (**D**–**F**) demonstrates percentages of IL‐18, IL‐18BP and IL‐18R expressing monocytes in leucocytes, respectively. (**G**) shows representative flow cytometric figures of MFI of IL‐18^+^ (i), IL‐18BP^+^ (ii) and IL‐18R^+^ (iii) monocyte. (**H**–**J**) shows mean fluorescent intensity (MFI) of IL‐18, IL‐18BP and IL‐18R expression in monocyte, respectively. Data are displayed as a boxplot for asthma patients (*n* = 31), and HC volunteers (*n* = 14), which indicates the median, interquartile range, the largest and smallest values for the number of volunteers indicated. *P* < 0.05 was taken as statistically significant. FMO = fluorescence minus one. ^‡^ *P* < 0.05 in comparison with HC group.

Little is known about the sources of increased plasma level of IL‐18 and expression level of IL‐18R on inflammatory cells in asthma. We therefore examined expression levels of IL‐18, IL‐18BP and IL‐18R in monocytes of asthmatic blood. The results showed that percentage of CD14^+^ monocytes in asthmatic blood was much greater than that in HC blood. The allergens tested had little effect on number of monocytes (Fig. 2A and B).

Approximately 8.2% and 7.3% of monocytes in HC and asthmatic blood expressed IL‐18, respectively. ASWE appeared to enhance IL‐18 expression in monocytes (Fig. 2C and D). More than 95% CD14^+^ monocytes in asthmatic or HC blood expressed IL‐18BP regardless of presence of allergens or not (Fig. 2C and E), which indicates 13‐fold more monocytes express IL‐18BP than IL‐18 in asthmatic blood. We also observed that percentage of IL‐18R^+^ monocytes was enhanced in asthmatic blood compared with that in HC blood. The allergens tested had little effect on IL‐18R expression on monocytes (Fig. 2C and F).

In terms of MFI (mean fluorescence intensity), ASWE and PPAE enhanced MFI of IL‐18 in asthmatic monocytes (Fig. 2Gi and H). On the other hand, asthmatic monocytes seemed to have less MFI of IL‐18BP than that in HC monocytes (Fig. 2Gii and I). The allergens tested had little influence on MFI of IL‐18R expressed in monocytes (Fig. 2Giii and J).

### Enhanced expression of IL‐18 and IL‐18R in asthmatic neutrophils

**Figure 3:**
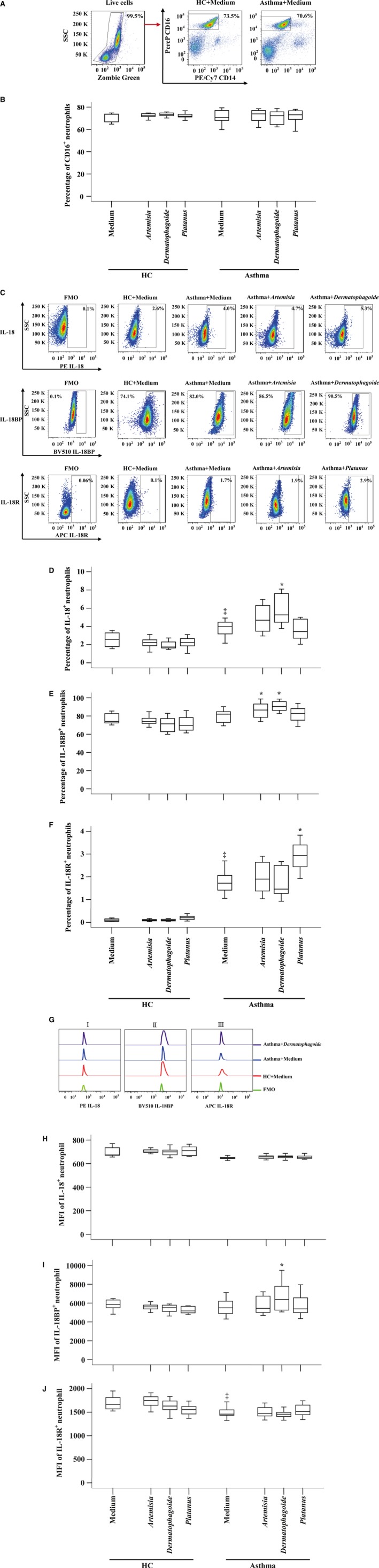
Expression of IL‐18, IL‐18BP and IL‐18 receptor (R) in peripheral blood CD16^+^ neutrophils of asthma patients and healthy control (HC) volunteers in the presence or absence of *Artemisia*,* Dermatophagoide* and *Platanus* allergens. (**A**) represents a gating strategy of CD16^+^ neutrophils in leucocytes. (**B**) shows proportion of CD16^+^ neutrophils in human peripheral blood leucocytes. (**C**) is a gating strategy of expression of IL‐18, IL‐18BP and IL‐18R in CD16^+^ neutrophils. (**D**–**F**) demonstrates percentages of IL‐18, IL‐18BP and IL‐18R expressing neutrophils in leucocytes, respectively. (**G**) shows representative flow cytometric figures of MFI of IL‐18^+^ (i), IL‐18BP^+^ (ii) and IL‐18R^+^ (iii) neutrophil. (**H**–**J**) show mean fluorescent intensity (MFI) of IL‐18, IL‐18BP and IL‐18R expression in neutrophil, respectively. Data are displayed as a boxplot for asthma patients (*n* = 31), and HC volunteers (*n* = 14), which indicates the median, interquartile range, the largest and smallest values for the number of volunteers indicated. *P* < 0.05 was taken as statistically significant. *P < 0.05 compared with medium control in asthma group. ^‡^ *P* < 0.05 compared with medium control in HC group. FMO = fluorescence minus one.

It has been reported that human neutrophil can release IL‐18 14, which can be a source of enhanced plasma IL‐18 of asthma patients. To further understand the involvement of IL‐18 in asthma, we examined expression of IL‐18, IL‐18BP and IL‐18R in peripheral blood neutrophils. The results showed that CD16^+^ neutrophils occupied approximately 73.5% and 70.6% leucocytes in HC and asthmatic blood (Fig. 3A and B). However, only 2.6% and 4.0% neutrophils expressed IL‐18 (Fig. 3C and D), and 0.1% and 1.7% neutrophils expressed IL‐18R in HC and asthmatic blood (Fig. 3C and F). On the other hand, more than 70% neutrophils in HC and asthmatic blood expressed IL‐18BP (Fig. 3C and E). We then worked out that approximately 17.5‐fold more neutrophils express IL‐18BP than IL‐18 in asthmatic blood. Moreover, ASWE and DAE increased proportions of IL‐18BP^+^ neutrophils when they were added to asthmatic blood (Fig. 3E). Asthmatic neutrophils seemed to have less MFI of IL‐18R than that in HC neutrophils (Fig. 3Giii and J). The allergens had little effect on MFI of IL‐18, IL‐18BP and IL‐18R in neutrophils (Fig. 3G–J).

### Enhanced expression of IL‐18 and IL‐18R in asthmatic B cells

**Figure 4:**
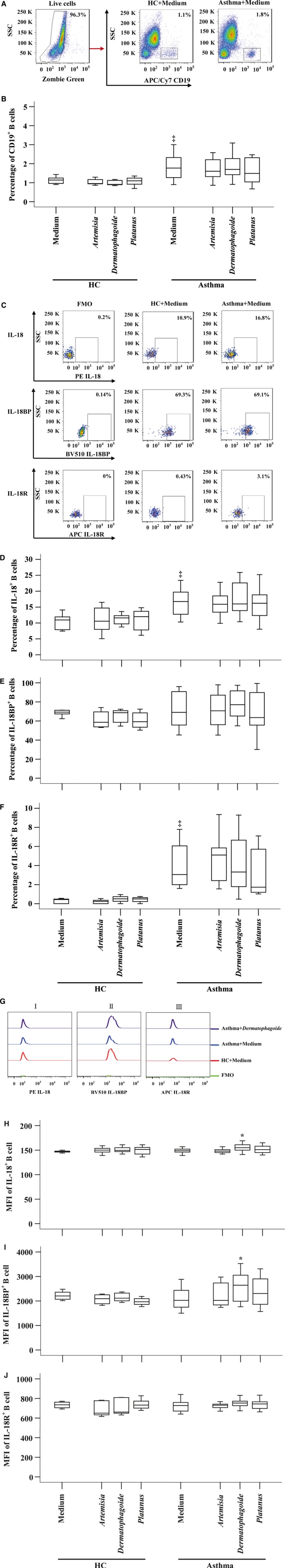
Expression of IL‐18, IL‐18BP and IL‐18 receptor (R) in peripheral blood CD19^+^ B cells of asthma patients and healthy control (HC) volunteers in the presence or absence of *Artemisia*,* Dermatophagoide* and *Platanus* allergens. (**A**) represents a gating strategy of CD19^+^ B cells in leucocytes. (**B**) shows proportion of CD19^+^ B cells in human peripheral blood leucocytes. (**C**) is a gating strategy of expression of IL‐18, IL‐18BP and IL‐18R in CD19^+^ B cells. (**D**–**F**) demonstrates percentages of IL‐18, IL‐18BP and IL‐18R expressing B cells in leucocytes, respectively. (**G**) shows representative flow cytometric figures of MFI of IL‐18^+^ (i), IL‐18BP^+^ (ii) and IL‐18R^+^ (iii) B cells. (**H**–**J**) shows mean fluorescent intensity (MFI) of IL‐18, IL‐18BP and IL‐18R expression in B cells, respectively. Data are displayed as a boxplot for asthma patients (*n* = 31), and HC volunteers (*n* = 14), which indicates the median, interquartile range, the largest and smallest values for the number of volunteers indicated. *P* < 0.05 was taken as statistically significant. ^*^ *P* < 0.05 compared with medium control in asthma group. ^‡^ *P* < 0.05 compared with medium control in HC group. FMO = fluorescence minus one.

Little is known about expression of IL‐18, IL‐18BP and IL‐18R in peripheral blood B cells, and we therefore examined expression of them in B cells of asthma patients. The results showed that proportion of CD19^+^ B cells increased in asthmatic blood compared with that in HC blood (Fig. 4A and B). Proportions of IL‐18^+^ (Fig. 4C and D) and IL‐18R^+^ (Fig. 4C and F) B cells were also increased in asthmatic blood in comparison with those in HC blood. Approximately 69.3% and 69.1% B cells in HC and asthmatic blood expressed IL‐18BP (Fig. 4C and E). It was observed that 4.1‐fold more B cells express IL‐18BP than IL‐18 in asthmatic blood. The allergens tested in this study did not alter proportions of IL‐18^+^, IL‐18BP^+^ and IL‐18R^+^ B cells in HC and asthmatic blood (Fig. 4D–F). However, DAE seemed to induce elevated MFI of IL‐18 (Fig. 4Gi and H) and IL‐18BP (Fig. 4Gii and I) in B cells of asthma patients.

### Enhanced IL‐18R^+^ monocytes, but reduced IL‐18BP^+^ monocytes in OVA‐sensitized mouse blood

**Figure 5:**
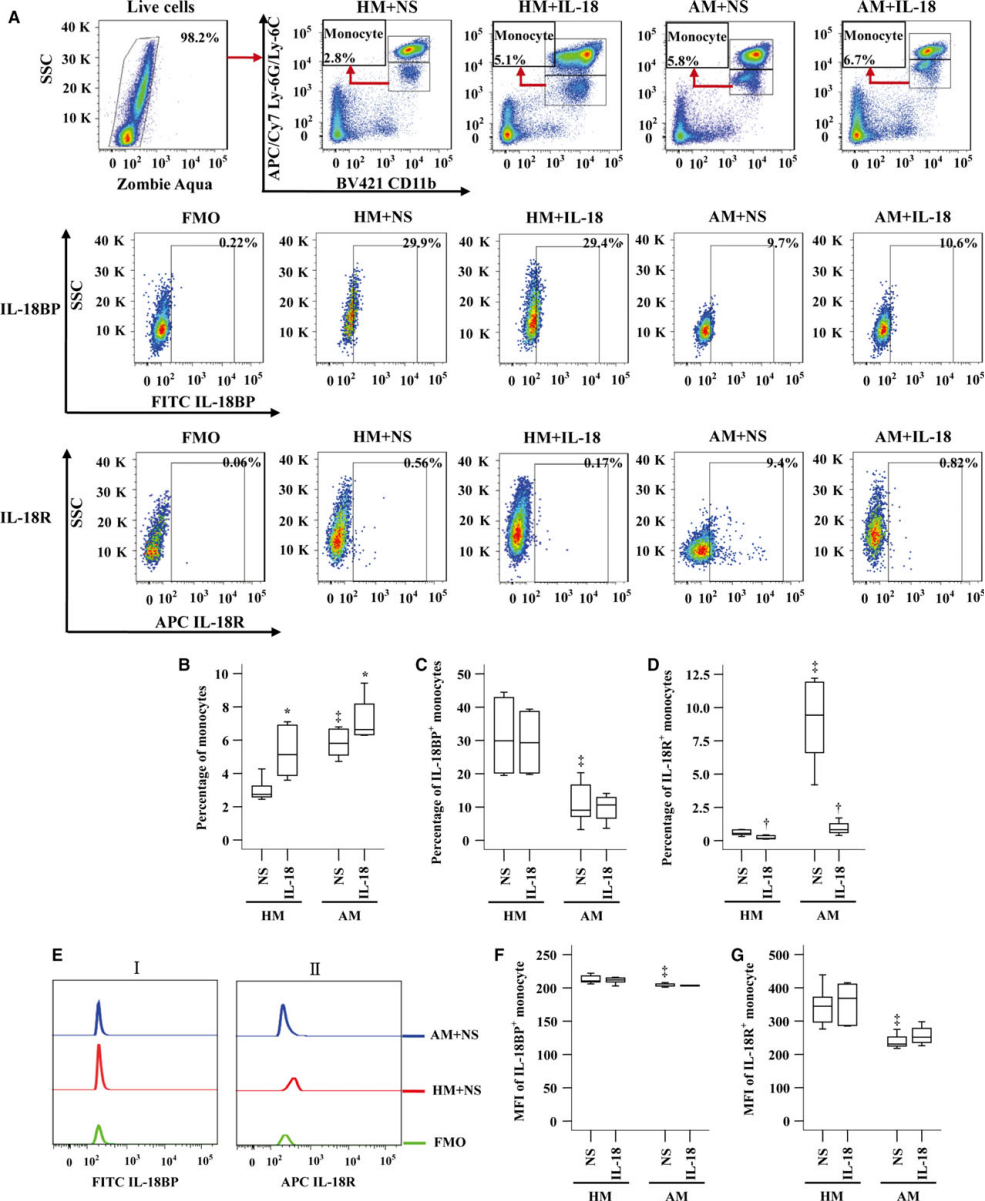
Expressions of IL‐18BP and IL‐18 receptor (R) in monocytes of sensitized or non‐sensitized mouse blood in the presence or absence of IL‐18. (**A**) represents a gating strategy of CD11b^+^ Ly‐6G/Ly‐6C ^low^ monocyte expression in mouse leucocytes, and gating strategies for IL‐18BP and IL‐18R expression in monocytes of mouse blood. (**B–D**) demonstrates changes in percentages of monocytes, and proportions of IL‐18BP and IL‐18R expressing monocytes out of leucocytes, respectively, in response to IL‐18 (10 ng/ml) or normal saline (NS). (**E**) shows representative flow cytometric figures of MFI of IL‐18BP^+^ (i) and IL‐18R^+^ (ii) monocyte. (**F** and **G**) shows changes in mean fluorescent intensity (MFI) of IL‐18BP and IL‐18R expression in monocyte, respectively, in response to IL‐18 or NS. Data are displayed as a boxplot for sensitized (AM, *n* = 7) and non‐sensitized (HM, *n* = 6–7) mice, which indicates the median, interquartile range, the largest and smallest values for the number of volunteers indicated. *P* < 0.05 was taken as statistically significant. ^*^ *P* < 0.05 increased expression compared with corresponding NS group, ^‡^ *P* < 0.05 in comparison with HM NS group, ^†^ *P* < 0.05 decreased expression compared with corresponding NS group. FMO = fluorescence minus one.

To understand further the influence of IL‐18 on monocytes, we investigated expression of IL‐18BP and IL‐18R in mouse monocytes with or without IL‐18 challenge. The results showed that the numbers of monocytes (Fig. 5A and B) and IL‐18R^+^ monocytes (Fig. 5A and D) were increased in OVA‐sensitized mouse blood. Sensitization reduced number of IL‐18BP^+^ monocytes (Fig. 5A and C). IL‐18 at 10 ng/ml enhanced number of monocytes by 87.2% and 14.4%, respectively (Fig. 5A and B), but eliminated IL‐18R^+^ monocytes (Fig. 5A and D) in blood of non‐sensitized and sensitized mice. OVA sensitization reduced MFI of IL‐18BP (Fig. 5Ei and F) and IL‐18R (Fig. 5Eii and G) on monocyte.

### Decreased IL‐18BP^+^ neutrophils, but increased IL‐18R^+^ neutrophils in OVA‐sensitized mouse blood

**Figure 6:**
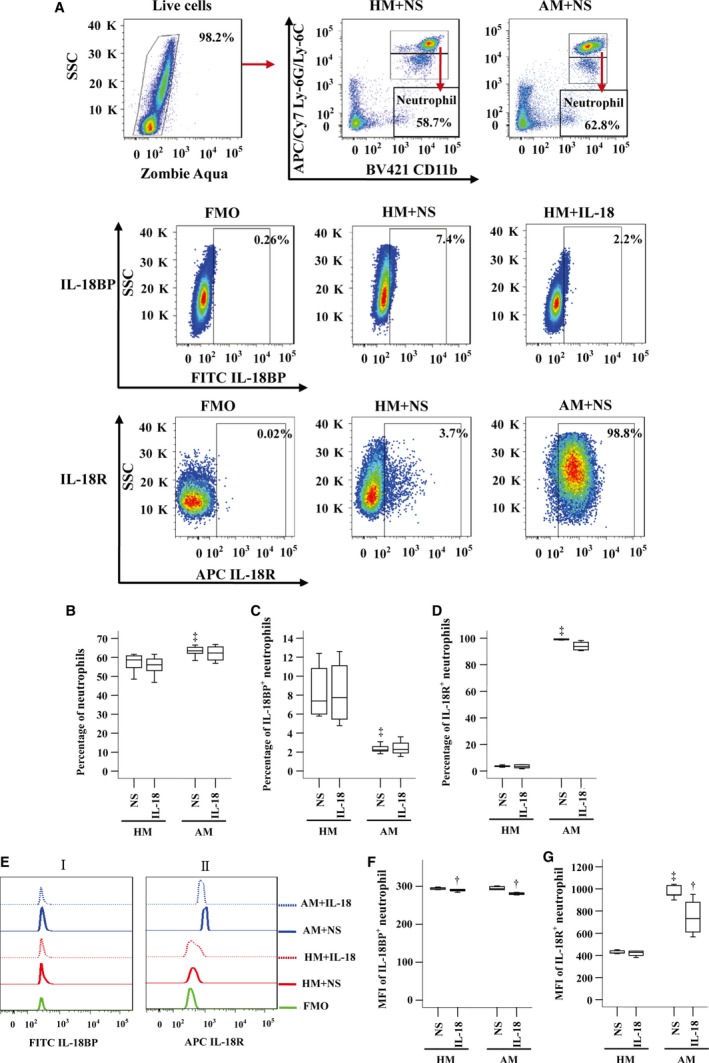
Expressions of IL‐18BP and IL‐18 receptor (R) in neutrophils of sensitized or non‐sensitized mouse blood in the presence or absence of IL‐18. (**A**) represents a gating strategy of CD11b^+^ Ly‐6G/Ly‐6C ^high^ neutrophil expression in mouse leucocytes, and gating strategies for IL‐18BP and IL‐18R expression in neutrophils of mouse blood. (**B**–**D**) demonstrates changes in percentages of neutrophils, and proportions of IL‐18BP and IL‐18R expressing neutrophils out of leucocytes, respectively, in response to IL‐18 (10 ng/ml) or normal saline (NS). (**E**) shows representative flow cytometric figures of MFI of IL‐18BP^+^ (i) and IL‐18R^+^ (ii) neutrophil. (**F** and **G**) shows changes in mean fluorescent intensity (MFI) of IL‐18BP and IL‐18R expression in neutrophil, respectively, in response to IL‐18 or NS. Data are displayed as a boxplot for sensitized (AM, *n* = 7) and non‐sensitized (HM, *n* = 6–7) mice, which indicates the median, interquartile range, the largest and smallest values for the number of volunteers indicated. *P* < 0.05 was taken as statistically significant. ^‡^ *P* < 0.05 compared NS in HM group, ^†^ *P* < 0.05 compared with corresponding NS group. FMO = fluorescence minus one.

We also investigated expression of IL‐18BP and IL‐18R in neutrophils of sensitized and non‐sensitized mice. The results showed that sensitization enhanced number of neutrophils in mouse blood (Fig. 6A and B). Compared with non‐sensitized mice, sensitized mice had decreased proportion of IL‐18BP^+^ neutrophils (Fig. 6A and C), but increased IL‐18R^+^ neutrophils (Fig. 6A and D) in their blood. Sensitization also enhanced MFI of IL‐18R on neutrophils (Fig. 6Eii and G). IL‐18 diminished MFI of IL‐18BP in neutrophils by 1.7% and 4.6% in non‐sensitized and sensitized mouse blood, respectively (Fig. 6Ei and F), and eliminated MFI of IL‐18R by 29.4% in sensitized mouse neutrophils (Fig. 6Eii and G).

### Enhanced IL‐18BP^+^ macrophages, but reduced IL‐18R^+^ macrophages in OVA‐sensitized mouse lung

**Figure 7:**
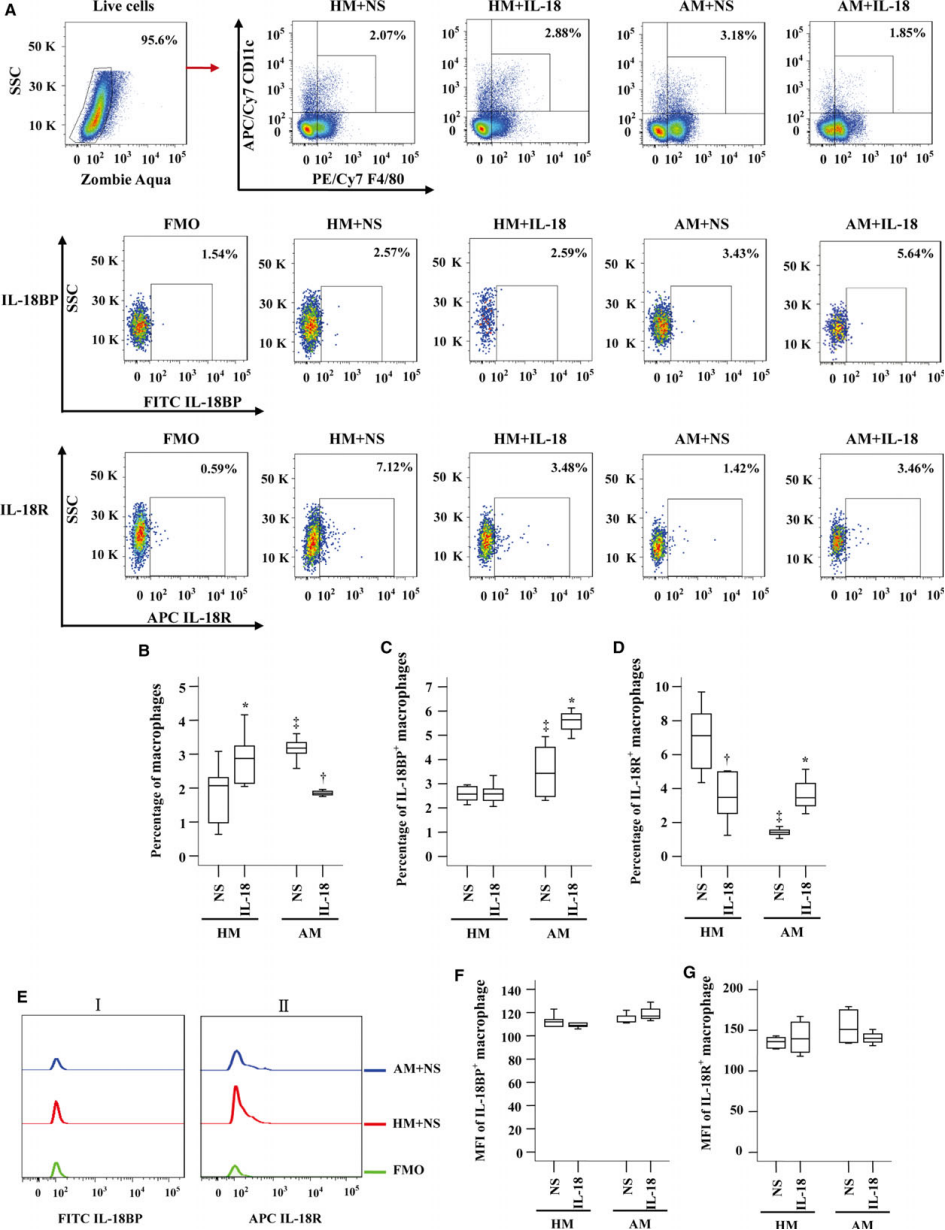
Expressions of IL‐18BP and IL‐18 receptor (R) in macrophags of sensitized or non‐sensitized mouse lung cells in the presence or absence of IL‐18. (**A**) represents a gating strategy of F4/80^+^ CD11c^+^ macrophage expression in dispersed lung cells of mice, and gating strategies for IL‐18BP and IL‐18R expression in macrophages of mouse lung cells. (**B**–**D**) demonstrates changes in percentages of macrophages, and proportions of IL‐18BP and IL‐18R expressing macrophages out of dispersed lung cells, respectively, in response to IL‐18 (10 ng/ml) or normal saline (NS). (**E**) shows representative flow cytometric figures of MFI of IL‐18BP^+^ (i) and IL‐18R^+^ (ii) macrophage. (**F** and **G**) show changes in mean fluorescent intensity (MFI) of IL‐18BP and IL‐18R expression in macrophage, respectively, in response to IL‐18 or NS. Data are displayed as a boxplot for sensitized (AM, *n* = 7) and non‐sensitized (HM, *n* = 6–7) mice, which indicates the median, interquartile range, the largest and smallest values for the number of volunteers indicated. *P* < 0.05 was taken as statistically significant. ^*^ *P* < 0.05 increased expression compared with corresponding NS group, ^‡^ *P* < 0.05 in comparison with HM NS group, ^†^ *P* < 0.05 decreased expression compared with corresponding NS group. FMO = fluorescence minus one.

As the influence of IL‐18 on expression of IL‐18BP and IL‐18R in macrophages remains unknown, we examined the issue in this study. The results showed that proportions of macrophages in dispersed lung cells were elevated in OVA‐sensitized mice (Fig. 7A and B). Sensitization also enlarged number of IL‐18BP^+^ macrophages (Fig. 7A and C), but reduced number of IL‐18R^+^ macrophages (Fig. 7A and D). IL‐18 at 10 ng/ml enhanced number of macrophages by 38.9% in non‐sensitized mice, but reduced percentage of macrophages by 41.8% in sensitized mice (Fig. 7A and B). IL‐18 up‐regulated expression of IL‐18BP (Fig. 7A and C) and IL‐18R (Fig. 7A and D) on lung macrophages by 64.4% and 144% in sensitized mice, respectively, but eliminated IL‐18R^+^ macrophages by 51.1% in non‐sensitized mice (Fig. 7A and D). IL‐18 had little effect on MFI of IL‐18BP (Fig. 7Ei and F) and IL‐18R (Fig. 7Eii and G) in mouse macrophage.

## Discussion

IL‐18 belongs to the IL‐1 family, which plays a major role in innate as well as acquired immunity. As IL‐18 stimulates T cells to produce increased IFN‐γ, IL‐13 and IL‐5 23, and IL‐13 and IL‐5 are potent Th2 cytokines, which play key pro‐inflammatory role in atopic asthma, it is believed that IL‐18 ought to be a causative factor for asthma. However, a study demonstrated that activation of the TLR2/NLRP3/IL‐18 axis can protect against asthma 3, complicating the role of IL‐18 in asthma. As IL‐18 carries out its biological functions mainly through its receptor IL‐18R 24, and IL‐18BP is a potent endogenous neutralizing antagonist of IL‐18, it is very likely that the role of IL‐18 in atopic asthma is decided by the balance between IL‐18, IL‐18BP and IL‐18R. We therefore examined expression of IL‐18, IL‐18BP and IL‐18R in parallel in inflammatory cells of asthma. Because monocytes/macrophages, neutrophils and B cells are involved in the pathogenesis of asthma, and they have close relationship with IL‐18, we first examined expression of IL‐18, IL‐18BP and IL‐18R in these cells.

We found for the first time that only 7.3% monocytes, 4.0% neutrophils and 16.8% B cells expressed IL‐18, and monocytes, neutrophils and B cells consist of approximately 2.3%, 70.6% and 1.8% leucocytes, which give 0.15%, 2.83% and 0.27% blood leucocytes are IL‐18^+^ monocytes, IL‐18^+^ neutrophils and IL‐18^+^ B cells, respectively, in asthmatic blood. Obviously, among the three cell types examined, neutrophils are predominant IL‐18 expressing leucocytes in asthmatic blood. In terms of density of IL‐18 expressed in these cells, MFIs of IL‐18 in monocytes, neutrophils and B cells of asthmatic blood are 712, 648 and 149, respectively, confirming that neutrophils are predominant IL‐18 expressing leucocytes in asthmatic blood.

We demonstrated for the first time that as much as 95% CD14^+^ monocytes, more than 70% neutrophils, and approximately 69.3% B cells in asthmatic or HC blood expressed IL‐18BP. This is an unexpected and striking finding as IL‐18BP is an endogenous antagonist with high neutralizing capacity in inhibition of action of IL‐18 9. A 13‐fold (95%/7.3%) more monocytes, 17.5‐fold (70%/4%) more neutrophils and 4.1‐fold (69.3%/17%) more B cells express IL‐18BP than IL‐18 indicate that there is excessive amount of IL‐18BP to completely abolish actions of IL‐18 in asthma. Indeed, the ratios between plasma concentrations of IL‐18BP and IL‐18 were 1 in HC volunteers and 12.8 in patients with asthma, supporting further the fact that there is excessive amount of IL‐18BP to completely abolish actions of IL‐18 in both HC volunteers and asthmatic patients. We therefore believe that actions of IL‐18 in asthma depend on the balance between IL‐18 and IL‐18BP. A report that a severe IL‐18/IL‐18BP imbalance results in Th1 lymphocyte and macrophage activation in patients with secondary haemophagocytic syndrome 25 may support the view that an IL‐18/IL‐18BP imbalance decides the role of IL‐18 in the pathogenesis of asthma.

The elevated plasma level of IL‐18BP in asthma patients indicates that excessive IL‐18BP is produced in the body. However, the expression of IL‐18BP by stimulated neutrophils, monocytes and B cells seems not to increase in asthmatic patients in comparison with healthy controls, which implicates that the excessive amount of plasma IL‐18BP is likely produced by certain cell types other than neutrophils, monocytes and B cells in the body.

We also discovered for the first time that approximately 64% IL‐18R^+^ monocytes, 1.7% IL‐18R^+^ neutrophils and 3.1% IL‐18R^+^ B cells are located in asthmatic blood. As for IL‐18BP, there should be 1.28% (64% × 2%) monocyte‐derived, 1.19% (1.7% × 70%) neutrophil‐derived and 0.045% (2.8% × 1.6%) B‐cell‐derived IL‐18R^+^ leucocytes in asthma blood. As a wide range of other cell types also expresses IL‐18R 8, it is very likely that huge number of IL‐18R expressing inflammatory cells is ready to react to IL‐18 in patients with asthma, suggesting the importance of IL‐18R in asthma.

We observed that ASWE and PPAE enhance IL‐18 expression in asthmatic monocytes; ASWE and DAE increase proportions of IL‐18BP and IL‐18 expression, and PPAE enhances IL‐18R expression in asthmatic neutrophils; DAE elevated MFI of IL‐18 and IL‐18BP in B cells of asthmatic blood. These results are a little confusing, but it implicates that allergens can enhance IL‐18, IL‐18BP and IL‐18R expression in its favourite cells in asthmatic blood.

In mouse model, OVA sensitization markedly reduced IL‐18BP^+^ monocytes and neutrophils, but enlarged number of IL‐18R^+^ monocytes and neutrophils in their blood, which implicates that sensitized mice should be more sensitive and responsive to IL‐18 stimulation than their human counterparts as a result of decreased IL‐18BP production and an enhanced IL‐18R expression. The finding that IL‐18 eliminated MFI of IL‐18R in mouse blood neutrophils and IL‐18R^+^ monocytes in mouse blood suggests that IL‐18 can down‐regulate expression of its own receptor, which may represent a self‐elimination mechanism of IL‐18, supporting the view that an IL‐18/IL‐18R imbalance decides the role of IL‐18 in the pathogenesis of asthma. The results that more than 78% human neutrophils in asthmatic blood, but <1.9% neutrophils in sensitized mouse blood expressed IL‐18BP; and that 1.7% human neutrophils in asthmatic blood, but more than 90% neutrophils in sensitized mouse blood expressed IL‐18R, which show clear difference between human and mouse asthmatic models. It is rather difficult to explain these discrepancies, but they may hint the controversial functions of murine and human leucocytes in allergic inflammation 26 and suggest once again the limitation of animal model in studying human diseases.

Induction of increased peripheral blood monocytes and lung macrophages by IL‐18 suggests that IL‐18 might promote monocytes to migrate and aggregate to local site and differentiate IL‐18BP^+^ macrophages during inflammatory process in mice. Although lack of direct evidence to prove IL‐18 can induce monocyte migration, the findings that IL‐18 administration can promote *in vivo* neutrophil 27, and mast cell accumulation 28 in mice may support our above anticipation.

It was found that proportion of macrophages in dispersed lung cells was elevated in OVA‐sensitized mice. However, IL‐18 appeared to reduce number of macrophages in lung of sensitized mice. The observations that sensitization increased number of IL‐18BP^+^ macrophages, and that IL‐18 up‐regulated expression of IL‐18BP in lung macrophages of sensitized mice indicate that certain allergen may eliminate contribution of IL‐18 to asthma *via* IL‐18BP^+^ macrophages. Moreover, the finding that sensitization reduced number of IL‐18R^+^ macrophages in lung further decreases the capability for IL‐18 contributing to development of asthma. As macrophages are the major source of IL‐18BP within the submucosa 29, it is not difficult to understand IL‐18 is unlikely to contribute to the pathogenesis of asthma through lung macrophages.

**Figure 8:**
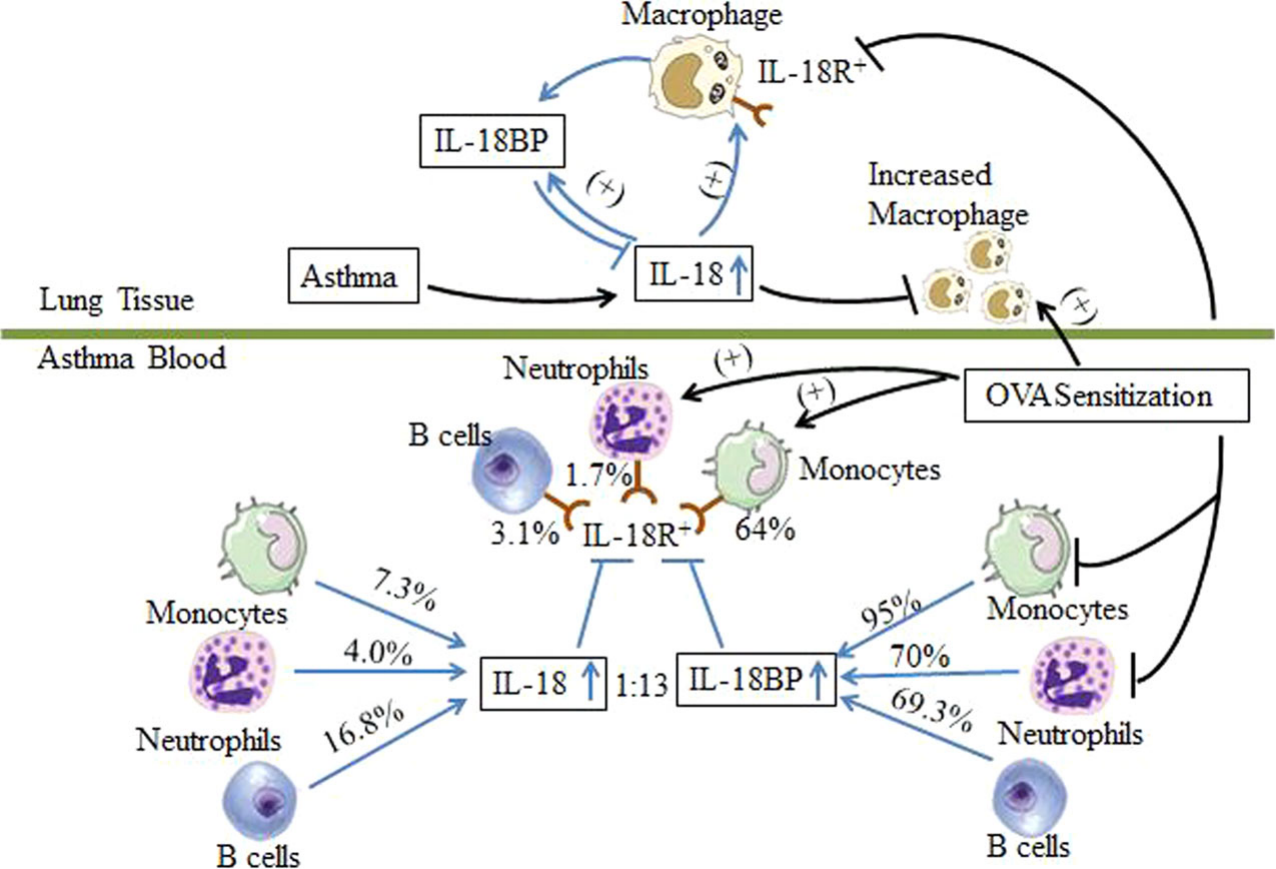
Summary graph of the role of IL‐18 in atopic asthma: the interactive balance between IL‐18, IL‐18 binding protein (BP) and IL‐18 receptor (R) decides the action of IL‐18 in asthma.

In conclusion, we found for the first time that enhanced expression of IL‐18R in monocytes, neutrophils and B cells in atopic asthma and that majority of these cell types express IL‐18BP. As IL‐18 can modulate IL‐18BP and IL‐18R expression, the role of IL‐18 in atopic asthma is very likely to be decided by the balance of IL‐18/IL‐18BP/IL‐18R expression in response to different allergens (Fig. 8). Therefore, IL‐18R blocking or IL‐18BP activity enhancing therapies may be useful for treatment of asthma.

## Authorship

HYZ carried out most experiments and wrote large part of the first draft of the manuscript. JLW and LW carried out the mouse sensitization and challenge tests, ELISA and generated part of the data and wrote a part of the first draft of the manuscript. HX and LPC performed the clinical study and wrote a part of the first draft of the manuscript. SHH designed and organized the study, analysed the data and wrote the second and final drafts of the manuscript. All authors read and approved the final manuscript.

## Conflict of interest

The authors confirm that there is no conflict of interests.

## References

[1] Rovina N , Dima E , Bakakos P , *et al* Low interleukin (IL)‐18 levels in sputum supernatants of patients with severe refractory asthma. Respir Med. 2015; 109: 580–7. 25840484 10.1016/j.rmed.2015.03.002

[2] Sanders NL , Mishra A . Role of interleukin‐18 in the pathophysiology of allergic diseases. Cytokine Growth Factor Rev. 2016; 32: 31–9. 27496752 10.1016/j.cytogfr.2016.07.001 PMC5124539

[3] Koch KN , Hartung ML , Urban S , *et al* Helicobacter urease‐induced activation of the TLR2/NLRP3/IL‐18 axis protects against asthma. J Clin Invest. 2015; 125: 3297–302. 26214524 10.1172/JCI79337 PMC4563744

[4] Oda H , Kawayama T , Imaoka H , *et al* Interleukin‐18 expression, CD8(+) T cells, and eosinophils in lungs of nonsmokers with fatal asthma. Ann Allergy Asthma Immunol. 2014; 112: 23–28 e1. 24331389 10.1016/j.anai.2013.09.004

[5] Ando M , Shima M . Serum interleukins 12 and 18 and immunoglobulin E concentrations and allergic symptoms in Japanese schoolchildren. J Investig Allergol Clin Immunol. 2007; 17: 14–9. 17323858

[6] Harada M , Obara K , Hirota T , *et al* A functional polymorphism in IL‐18 is associated with severity of bronchial asthma. Am J Respir Crit Care Med. 2009; 180: 1048–55. 19745201 10.1164/rccm.200905-0652OC

[7] Xu D , Chan WL , Leung BP , *et al* Selective expression and functions of interleukin 18 receptor on T helper (Th) type 1 but not Th2 cells. J Exp Med. 1998; 188: 1485–92. 9782125 10.1084/jem.188.8.1485 PMC2213413

[8] Sims JE , Smith DE . The IL‐1 family: regulators of immunity. Nat Rev Immunol. 2010; 10: 89–102. 20081871 10.1038/nri2691

[9] Novick D , Kim SH , Fantuzzi G , *et al* Interleukin‐18 binding protein: a novel modulator of the Th1 cytokine response. Immunity. 1999; 10: 127–36. 10023777 10.1016/s1074-7613(00)80013-8

[10] Kim SH , Eisenstein M , Reznikov L , *et al* Structural requirements of six naturally occurring isoforms of the IL‐18 binding protein to inhibit IL‐18. Proc Natl Acad Sci U S A. 2000; 97: 1190–5. 10655506 10.1073/pnas.97.3.1190 PMC15564

[11] Murai H , Qi H , Choudhury B , *et al* Alternaria‐induced release of IL‐18 from damaged airway epithelial cells: an NF‐kappaB dependent mechanism of Th2 differentiation? PLoS ONE. 2012; 7: e30280. 22347372 10.1371/journal.pone.0030280 PMC3274547

[12] Sawada M , Kawayama T , Imaoka H , *et al* IL‐18 induces airway hyperresponsiveness and pulmonary inflammation *via* CD4 + T cell and IL‐13. PLoS ONE. 2013; 8: e54623. 23382928 10.1371/journal.pone.0054623 PMC3558507

[13] Ye Q , Nakamura S , Sarria R , *et al* Interleukin 12, interleukin 18, and tumor necrosis factor alpha release by alveolar macrophages: acute and chronic hypersensitivity pneumonitis. Ann Allergy Asthma Immunol. 2009; 102: 149–54. 19230467 10.1016/S1081-1206(10)60246-3

[14] Silliman CC , Kelher MR , Gamboni‐Robertson F , *et al* Tumor necrosis factor‐alpha causes release of cytosolic interleukin‐18 from human neutrophils. Am J Physiol Cell Physiol. 2010; 298: C714–24. 19907017 10.1152/ajpcell.00011.2009 PMC2838563

[15] Lapointe TK , Buret AG . Interleukin‐18 facilitates neutrophil transmigration *via* myosin light chain kinase‐dependent disruption of occludin, without altering epithelial permeability. Am J Physiol Gastrointest Liver Physiol. 2012; 302: G343–51. 22135309 10.1152/ajpgi.00202.2011

[16] Hart G , Flaishon L , Shachar I . IL‐12 and IL‐18 down‐regulate B cell migration in an Ly49D‐dependent manner. Eur J Immunol. 2007; 37: 1996–2007. 17557376 10.1002/eji.200737083

[17] Pages F , Galon J , Karaschuk G , *et al* Epstein‐Barr virus nuclear antigen 2 induces interleukin‐18 receptor expression in B cells. Blood. 2005; 105: 1632–9. 15498855 10.1182/blood-2004-08-3196

[18] Von Mutius E . Presentation of new GINA guidelines for paediatrics. The Global Initiative on Asthma. Clin Exp Allergy. 2000; 30(Suppl 1): 6–10. 10849467 10.1046/j.1365-2222.2000.00089.x

[19] Akabane S , Matsuzaki K , Yamashita S , *et al* Constitutive activation of PINK1 protein leads to proteasome‐mediated and non‐apoptotic cell death independently of mitochondrial autophagy. J Biol Chem. 2016; 291: 16162–74. 27302064 10.1074/jbc.M116.714923 PMC4965565

[20] He S , Gaca MD , Walls AF . A role for tryptase in the activation of human mast cells: modulation of histamine release by tryptase and inhibitors of tryptase. J Pharmacol Exp Ther. 1998; 286: 289–97. 9655871

[21] Vom Berg J , Vrohlings M , Haller S , *et al* Intratumoral IL‐12 combined with CTLA‐4 blockade elicits T cell‐mediated glioma rejection. J Exp Med. 2013; 210: 2803–11. 24277150 10.1084/jem.20130678 PMC3865478

[22] Mukai K , BenBarak MJ , Tachibana M , *et al* Critical role of P1‐Runx1 in mouse basophil development. Blood. 2012; 120: 76–85. 22611151 10.1182/blood-2011-12-399113 PMC3390962

[23] Blom L , Poulsen LK . IL‐1 family members IL‐18 and IL‐33 upregulate the inflammatory potential of differentiated human Th1 and Th2 cultures. J Immunol. 2012; 189: 4331–7. 23028054 10.4049/jimmunol.1103685

[24] Wang Y , Hu C , Wang Z , *et al* Serum IL‐1beta and IL‐18 correlate with ESR and CRP in multidrug‐resistant tuberculosis patients. J Biomed Res. 2015; 29: 426–8. 26445573 10.7555/JBR.29.20150077 PMC4585440

[25] Mazodier K , Marin V , Novick D , *et al* Severe imbalance of IL‐18/IL‐18BP in patients with secondary hemophagocytic syndrome. Blood. 2005; 106: 3483–9. 16020503 10.1182/blood-2005-05-1980 PMC1895045

[26] Siracusa MC , Kim BS , Spergel JM , *et al* Basophils and allergic inflammation. J Allergy Clin Immunol. 2013; 132: 789–801; quiz 788. 24075190 10.1016/j.jaci.2013.07.046 PMC3903395

[27] Netea MG , Fantuzzi G , Kullberg BJ , *et al* Neutralization of IL‐18 reduces neutrophil tissue accumulation and protects mice against lethal Escherichia coli and Salmonella typhimurium endotoxemia. J Immunol. 2000; 164: 2644–9. 10679104 10.4049/jimmunol.164.5.2644

[28] Wang J , Zhang H , Zheng W , *et al* Correlation of IL‐18 with tryptase in atopic asthma and induction of mast cell accumulation by IL‐18. Mediators Inflamm. 2016; 2016: 4743176. 27069315 10.1155/2016/4743176 PMC4812453

[29] Corbaz A , ten Hove T , Herren S , *et al* IL‐18‐binding protein expression by endothelial cells and macrophages is up‐regulated during active Crohn's disease. J Immunol. 2002; 168: 3608–16. 11907126 10.4049/jimmunol.168.7.3608

